# Applicability of drug-related problem (DRP) classification system for classifying severe medication errors

**DOI:** 10.1186/s12913-023-09763-3

**Published:** 2023-07-10

**Authors:** Carita Linden-Lahti, Anna Takala, Anna-Riia Holmström, Marja Airaksinen

**Affiliations:** 1 Present Address: Division of Pharmacology and Pharmacotherapy, Faculty of Pharmacy, University of Helsinki, Viikinkaari 5 E, 00014 Helsinki, Finland; 2 HUS Pharmacy, Helsinki University Hospital, Stenbäckinkatu 9B, 00029 HUS, Helsinki, Finland

**Keywords:** Medication safety, Medication error, Severe medication error, Drug-related problem, Taxonomy, Classification system

## Abstract

**Background:**

Several classification systems for medication errors (MEs) have been established over time, but none of them apply optimally for classifying severe MEs. In severe MEs, recognizing the causes of the error is essential for error prevention and risk management. Therefore, this study focuses on exploring the applicability of a cause-based DRP classification system for classifying severe MEs and their causes.

**Methods:**

This was a retrospective document analysis study on medication-related complaints and authoritative statements investigated by the Finnish National Supervisory Authority for Welfare and Health (Valvira) in 2013–2017. The data was classified by applying a previously developed aggregated DRP classification system by Basger et al. Error setting and harm to the patient were identified using qualitative content analysis to describe the characteristics of the MEs in the data. The systems approach to human error, error prevention, and risk management was used as a theoretical framework.

**Results:**

Fifty-eight of the complaints and authoritative statements concerned MEs, which had occurred in a wide range of social and healthcare settings. More than half of the ME cases (52%, *n* = 30) had caused the patient’s death or severe harm. In total, 100 MEs were identified from the ME case reports. In 53% (*n* = 31) of the cases, more than one ME was identified, and the mean number of MEs identified was 1.7 per case. It was possible to classify all MEs according to aggregated DRP system, and only a small proportion (8%, *n* = 8) were classified in the category “Other,” indicating that the cause of the ME could not be classified to specific cause-based category. MEs in the “Other” category included dispensing errors, documenting errors, prescribing error, and a near miss.

**Conclusions:**

Our study provides promising preliminary results for using DRP classification system for classifying and analyzing especially severe MEs. With Basger et al.’s aggregated DRP classification system, we were able to categorize both the ME and its cause. More research is encouraged with other ME incident data from different reporting systems to confirm our results.

## Background

Medication errors (MEs) are one of the major concerns in patient safety [1–3]. A medication error is any preventable event that may cause or lead to inappropriate medication use or patient harm [4]. There is a need to learn effectively from errors that have already happened using error reporting systems and other available data sources to improve medication safety [1, 5–8]. Especially the reoccurrence of MEs causing severe harm or even the death of the patient should be prevented, which is also promoted by global actions [2].

In ME reporting systems and studies on medication safety, data have been typically analyzed using error classification systems and taxonomies. Theoretical or conceptual frameworks have usually guided the development of error classification systems, or the process has been data-driven [9]. Depending on the aim of the classification, the system can be contextual (e.g., the place or the medicine involved), modal (how the error happened, e.g., omission), or psychological (why the error occurred, e.g., skill-based error) [10]. Classification systems and taxonomies need to relate to similar events, describe essential clinical and systemic factors, and support analysis purposes [11].

Although there are multiple taxonomies for MEs, they often share the limitation of being in too general level and describing only outcomes, not the causes of the errors [12]. This deficiency has been noted in many widely internationally used classification systems (e.g., World Health Organization, National Coordinating Council for Medication Error Reporting and Prevention) [13, 14], but the challenges to describing MEs accurately enough and with the causes and contributing factors are still partly unresolved. Without knowing the causes and contributing factors of MEs, essential information for error prevention and risk management are lacking [12, 15]. Adequate information on what happened and why is crucial, especially when preventing severe MEs [16–18]. A classification system that gives enough information for medication error prevention and risk management, is desirable in the system-based approach to medication safety [11, 12]. Consequently, there is a need to develop ME classification systems further to support system-based medication safety.

Exploring the utility of existing classification systems, including also causes, can provide an option for acquiring more information about MEs to find novel and robust methods for describing ME data. Previous research has described the application of adverse drug event (ADE) classification systems to MEs, but the evidence is still limited [15]. According to our knowledge, none of the studies has evaluated how classification systems for drug-related problems (DRPs) could be applied to ME data, even though many of them also include causes for the problems [19].

## Material and methods

### Definitions of the key concepts used in this study

**Fig. 1:**
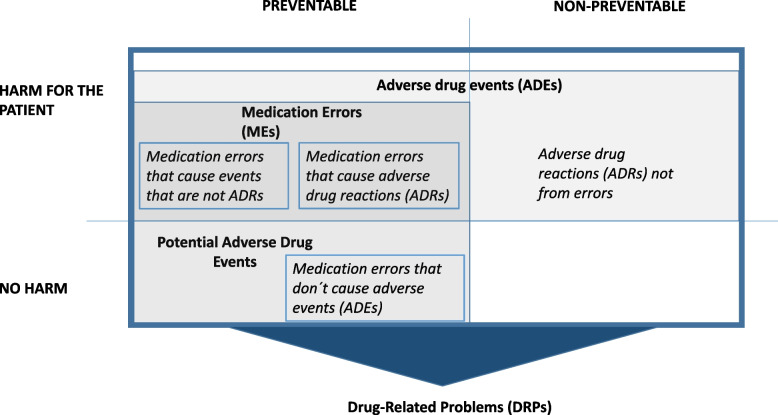
The relationship between the definitions of drug-related problems, adverse drug events, and medication errors summarized and modified from [4, 12, 20, 22–26]

Drug-related problem (DRP) is an event or circumstance involving drug therapy that actually or potentially interferes with desired outcomes (e.g., the effect of the drug treatment is not optimal, Table 1) [20]. DRPs occur or have the potential to happen, because of a prior event or events that perform as a cause or causes of the DRP (e.g., failures associated with the medication use process) [20, 21]. As MEs can cause DRPs (e.g., error in prescribing, Table 1 and Fig. 1), this connection supports using existing DRP classification systems for classifying MEs. Table 1 and Fig. 1 present the relationship between the definitions of ADEs, DRPs and MEs as used in this study.

**Table 1:** Key definitions of drug-related problems (DRPs), adverse drug events (ADEs), adverse drug reactions (ADRs), and medication errors (MEs) as used in this study

**Drug-related problem (DRP)**	An event or circumstance involving drug therapy that actually or potentially interferes with desired health outcomes [20]. Drug-related problems can be caused by medication errors, but there might be no error involved. A medication error does not necessarily lead to a drug-related problem; there can be no problem or a potential problem
**Adverse Drug Event (ADE)**	Any injuries due to medication [22, 23]. ADEs includes injuries caused by medication errors, adverse drug reactions, allergic reactions, and overdoses
**Potential Adverse Drug Event**	*Definition 1:* Those adverse drug events that did not cause an injury to a patient, but which had the potential to harm [24] *Definition 2:* A potential adverse drug event is a medication error with the potential to cause an injury, but which does not actually cause any injury, either because of specific circumstances, chance, or because the error is intercepted and corrected [23]
**Medication Error (ME)**	*Definition 1*: A medication error is a failure in the treatment process that leads to, or has the potential to lead to, harm to the patient [12] *Definition 2*: It is any preventable event that may cause or lead to inappropriate medication use or patient harm while the medication is in the control of the healthcare professional, patient, or consumer [4]. Such events may be related to professional practice, healthcare products, procedures, and systems, including prescribing, order communication, product labeling, packaging, and nomenclature, compounding, dispensing, distribution, administration, education, monitoring, and use *Definition 3*: A medication error is an unintended failure in the drug treatment process that leads to, or has the potential to lead to, harm to the patient [25]
**Adverse Drug Reactions (ADR)**	Noxious and unintended effects resulting not only from the authorized use of a medicinal product at normal doses, but also from medication errors [12, 26]

### Aim of the study

This study is to explore the applicability of a cause-based DRP classification system in classifying severe MEs and their causes.

### Study design and setting

This was a retrospective document analysis study [27] on medication-related complaints and authoritative statements investigated by the Finnish National Supervisory Authority for Welfare and Health (Valvira). Valvira is a national authority investigating patient safety incidents that have led to the severe harm or death of a patient due to inappropriate care [28]. Thus, Valvira has unique and extensive data emphasized on severe MEs that may rarely be captured by medication error reporting systems [29]. Contrary majority of the typical ME register data that mainly consist of structured data, Valvira´ s investigational documentation is based on qualitative descriptions of the incidents. The investigations are based on complaints made by patients and/or their relatives, notifications by healthcare organizations, or statement requests made by other Finnish authorities (e.g., the police, the parliamentary ombudsman, and the courts). The annual number of all new complaints and statement requests from social and healthcare was 632 in 2022 [30].

The theoretical framework for the study was the systems approach to human error, error prevention, and risk management through understanding the processes and causes leading to severe MEs [31, 32].

### Material

**Fig. 2:**
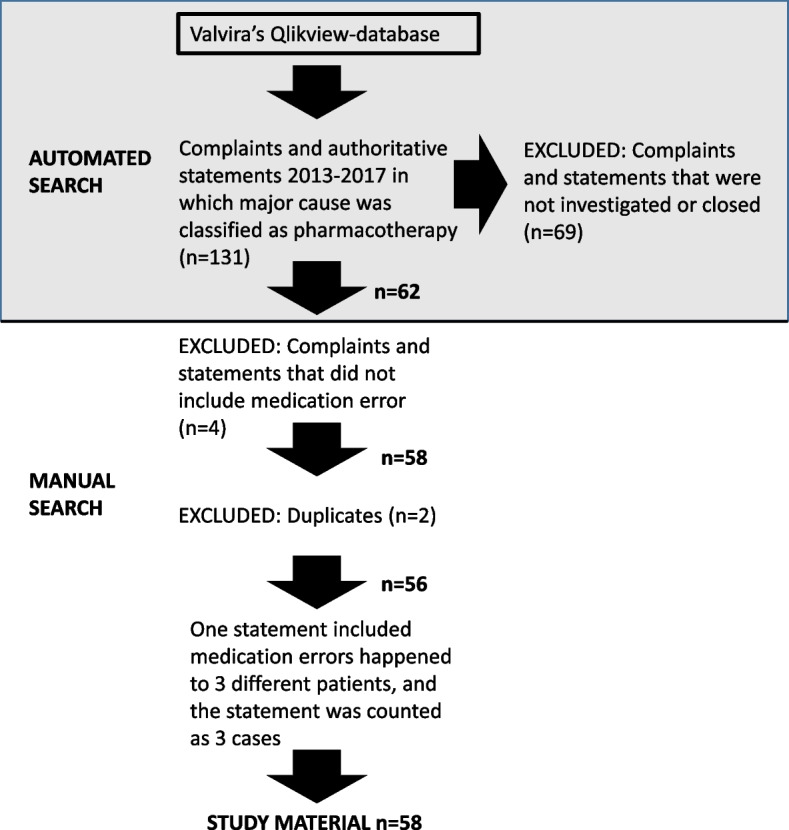
The protocol for obtaining the research data on the medication-related complaints and authoritative statements from 2013–2017 in which the major causes were classified as pharmacotherapy by the National Supervisory Authority for Welfare and Health (Valvira)

The research data consisted of all medication-related complaints and authoritative statements that Valvira had investigated and concluded from 2013–2017. The following inclusion criteria were used for getting the research data: 1) the primary cause of the complaint or statement was classified as “pharmacotherapy” by Valvira; 2) Valvira assessed the case to involve inappropriate patient care (error in medication), and 3) the case was closed (Fig. 2). The search of the document material was first performed as an automatic search in Valvira´s electronic database (Qlikview) and then finalized by a manual search by one of the researchers (AT) to ensure that all the automatically searched cases fulfilled the study inclusion criteria.

Valvira´s documentation for the complaints and authoritative statements typically contained: 1) a copy of the patient records and other documents needed for incident evaluation; 2) responses from the professionals involved in the case and/or the managers of the healthcare organization; 3) an external expert (physician or another specialist) statement; and 4) the incident report by Valvira’s Senior Medical or Legal Officer. All the incident documentation was qualitative data in nature. Some of the incident documentation was in narrative form, and it described the incident and its circumstances, as well as the conclusion of the case. The total written material per case varied between 20–150 pages.

### Research method

Several DRP classification systems have been established over time [19]. One important feature of the DRP classification systems is that they should be able to differentiate DRPs and their causes [21]. The present study applied the newest comprehensive DRP classification system, which was aggregated by Basger et al. based on a systematic inventory of existing DRP classification systems [19, 21]. Their classification system is comprehensive, easy to use, and separates DRPs and their causes. It forms a hierarchical classification system consisting of nine cause-of-DRP categories, including 33 subcategories and 58 sub-subcategories (Table 2). Basger et al.’s [21] aggregated classification system was adopted as such for the present study.

**Table 2:** Aggregated classification system for causes of DRPs created by Basger et al. [21]. MEs found in the Valvira’s data (*n* = 100) are presented as bolded

Aggregated System Category	Aggregated System Subcategory	Aggregated System Sub-subcategory
**1. Drug selection (*n* = 22)**	**1.1 Inappropriate drug due to contraindication, ineffectiveness, regimen (regular rather than “when required”) or safer alternative available (*n* = 7)**	**1.1.1 Precaution with the use of this drug (*n* = 1)** **1.1.2. Drug (absolutely) contraindicated (*n* = 5)** 1.1.3. An unnecessary drug is taken because of the use of another drug **1.1.4. Drug is not the most safe/effective treatment for the patient’s medical condition according to guidelines (*n* = 1)** 1.1.5. Drug is not effective for the indication being treated 1.1.6. Medical condition is refractory to drug
	**1.2 No indication for drug (*n* = 4)**	**1.2.1. No (documented) indication apparent (*n* = 4)** 1.2.2. No indication due to duplication 1.2.3. Indication does not warrant drug treatment
	**1.3 Inappropriate combination of drugs, or** **drugs and food, or drugs and alcohol (*n* = 4)**	**1.3.1. A drug interaction may cause/causes an undesirable reaction by increasing the therapeutic effect of one or both drugs (*n* = 3)** **1.3.2. A drug interaction may cause/causes an undesirable reaction by decreasing the therapeutic effect of one or both drugs (*n* = 1)** 1.3.3. A drug interaction may cause/causes a hypersensitivity reaction
	1.4 Indication not treated/missing therapy	(no sub-subcategory)
	1.5 More cost-effective drug available	(no sub-subcategory)
	**1.6 Synergistic/preventive drug required and** **not given (*n* = 7)**	**1.6.1. Preventive drug therapy is required to reduce the risk of developing a new condition (*n* = 2)** **1.6.2. A medical condition requires additional pharmacotherapy to attain synergistic or additive effects (*n* = 5)**
**2. Drug form (*n* = 1)**	**2.1 Inappropriate or suboptimal drug form (*n* = 1)**	(no sub-subcategory)
**3. Dose selection (*n* = 13)**	**3.1 Drug dose too low (*n* = 1)**	(no sub-subcategory)
	**3.2 Drug dose too high (*n* = 8)**	(no sub-subcategory)
	3.3 Dosage regimen not frequent enough	(no sub-subcategory)
	3.4 Dosage regimen too frequent	(no sub-subcategory)
	**3.5 Deterioration/improvement of disease** **state requiring dosage adjustment (*n* = 3)**	**3.5.1. Deterioration of disease state requiring dosage adjustment (*n* = 3)** 3.5.2. Improvement of disease state requiring dosage adjustment
	**3.6 Dosage instructions unclear, incomplete,** **or not understood by patient/carer (*n* = 1)**	(no sub-subcategory)
**4. Treatment duration (*n* = 11)**	**4.1 Duration of treatment too short (*n* = 7)**	(no sub-subcategory)
	**4.2 Duration of treatment too long (*n* = 4)**	(no sub-subcategory)
**5. Drug use process (*n* = 18)**	**5.1 Inappropriate timing of administration and/or dosing intervals by patient/carer/nurse (*n* = 5)**	(no sub-subcategory)
	**5.2 Drug underused/underadministered (*n* = 4)**	5.2.1. The patient chose to take the wrong dose, which was lower than prescribed 5.2.2. The patient chose to take a drug on a “when required” basis rather than on a regular basis 5.2.3. The patient misunderstood the directions 5.2.4. The patient felt better or worse 5.2.5. The patient had a fear of adverse effects 5.2.6. The patient did not believe the drug was effective/believed the drug was toxic 5.2.7. The patient occasionally forgot to take the drug **5.2.8. The drug was underadministered by the health care professional/carer (*n* = 4)**
	**5.3 Drug overused/overadministered (*n* = 3)**	5.3.1. The patient chose to take the wrong dose, which was higher than prescribed 5.3.2. The patient misunderstood the directions 5.3.3. The patient forgot they had already taken the drug **5.3.4. The drug was overadministered by carer or health care professional (*n* = 3)**
	5.4 Drug not taken/administered at all	5.4.1. The patient chose to discontinue a drug by choice or for an illogical or irrational reason 5.4.2. Patient forgot to take the drug
	5.5 Wrong drug selected, taken, or administered	(no sub-subcategory)
	5.6 Drug abused	(no sub-subcategory)
	**5.7 Patient, carer, or nurse unable to use/does** **not use drug/form as directed (*n* = 1)**	5.7.1. Patient uses drug incorrectly through difficulty or ignorance **5.7.2. Patient barriers are present (*n* = 1)**
	**5.8 Adequate information not provided or** **not understood or misunderstood or not** **followed (*n* = 5)**	**5.8.1. Adequate information about drug not provided (*n* = 3)** 5.8.2. Incorrect information about drug provided **5.8.3. Adequate information about disease state management not provided or not understood or not followed (*n* = 2)** 5.8.4. Incorrect information provided about disease state
	5.9 Patient uses or stores drug inappropriately	5.9.1. Inappropriate use/storage 5.9.2. Stockpiling
**6. Logistics (*n* = 11)**	**6.1 Prescribed drug not available (*n* = 1)**	6.1.1. The patient/carer had difficulties obtaining the drug **6.1.2. A drug has been discontinued, is not on formulary, or is out-of-stock (*n* = 1)** 6.1.3. A drug order does not meet legislative requirements
	**6.2 Drug order incorrect, incomplete, poorly** **legible/illegible or discrepant (also known** **as transferring error) (*n* = 2)**	6.2.1. A drug order is incorrect or incomplete **6.2.2. Drug order/transition of care discrepancy (*n* = 2)** 6.2.3. The way in which information/directions were written caused the patient/carer to misuse the drug
	**6.3 Error in drug selection (*n* = 8)**	6.3.1. Doctor chooses the wrong drug 6.3.2. Pharmacist selects the wrong/expired drug from dispensary shelf 6.3.3. Nurse administers drug from the wrong patient’s drug chart **6.3.4. Patient takes someone else’s drug (*n* = 8)**
**7. Monitoring (*n* = 14)**	7.1 Monitoring too frequent	7.1.1. Monitoring of disease state too frequent 7.1.2. Therapeutic drug monitoring too frequent 7.1.3. Monitoring for the effect/adverse effect of drug too frequent
	**7.2 No or too infrequent monitoring (*n* = 14)**	**7.2.1. Monitoring of disease state absent or too infrequent (*n* = 4)** **7.2.2. Therapeutic drug monitoring absent or too infrequent (*n* = 5)** **7.2.3. Monitoring for the effect/adverse effect of drug absent or too infrequent (*n* = 5)** 7.2.4. Monitoring may have occurred but is unavailable or not documented
	7.3 Inappropriate test ordered	(no sub-subcategory)
	7.4 Patient unable to attend/pay for monitoring	(no sub-subcategory)
**8. Unexpected or adverse drug reaction or no obvious cause of DRP (*n* = 2)**	**8.1 An adverse drug reaction occurred (*n* = 2)**	8.1.1. A drug causes an undesirable reaction that is not dose related **8.1.2. A drug causes an undesirable reaction at normal therapeutic dose (*n* = 2)** 8.1.3. Allergic drug reaction
	8.2 No obvious cause of treatment failure	(no sub-subcategory)
**9. Other: A cause that cannot be classified into one of the 8 categories (*n* = 8)**		

MEs were identified and classified from Valvira’s data by the main researcher (AT). First, the qualitative and partly narrative data in Valvira’s documentation was carefully read case by case. Identified ME process and contributing factors were summarized as a brief anonymized case description for each case. MEs were categorized from these brief case descriptions using Basger et al.’s. aggregated DRP classification system [21]. In cases where any difficulties were encountered in the categorization, another researcher was consulted (CLL), and the final classification was decided by a consensus from two researchers. In cases where no suitable category was found for the ME, the error was classified to the "Other” category (a cause that cannot be classified into any other categories, Category 9 in Table 2). The main researcher made notes of those MEs for detecting possible missing categories in the aggregated DRP classification system. Because severe MEs are often complex processes including several errors [16, 17, 31, 32], all identified MEs were categorized from description of each case.

Error setting and harm to the patient were identified and documented for the data analysis to explain the essential characteristics of the MEs in the data. Four categories were used to classify the severity of harm to the patient: death, severe harm, non-severe harm, and no harm. Harm to the patient was defined as severe when the error had been life-threatening, led to hospitalization or prolonged hospitalization, or caused permanent or significant injury with incapacity [33]. The data categorized in this study were quantitatively analyzed in Microsoft Excel for descriptive statistics (frequencies and percentages).

## Results

Valvira received a total of 1654 complaints and statement requests from 2013–2017. Of these cases, 58 cases were medication-related and included in this study. The identified MEs had occurred in a wide range of social and healthcare settings, most commonly in assisted living facilities (*n* = 16, 25% of all settings, Table 3). There were 6 cases (10%) where more than one care setting was involved in the error process. In more than half of the cases (52%, *n* = 30), the ME caused the patient’s death or severe harm. Other characteristics of the data have been published elsewhere [17].

**Table 3:** Error settings and severity of harm caused by the MEs identified from Valvira’s data from 2013–2017 (*n* = 58)

Characteristic	N	%
**Error setting**^**a**^	**64**	100
Assisted living facility	16	25
University hospital (secondary and tertiary care)	10	16
Primary care ward (inpatient care outside the hospital)	10	16
Central hospital (secondary care)	10	16
Primary care hospital	9	14
Public health center (outpatient care)	4	6
Home care	3	5
Community pharmacy	1	2
Private medical center (outpatient care)	1	2
**Severity of harm**	**58**	100
Death	21	36
Severe harm	9	16
Non-severe harm	19	33
No harm	3	5
Not able to assess	6	10

^a^It was possible that the ME case had occurred in more than one care setting

**Fig. 3:**
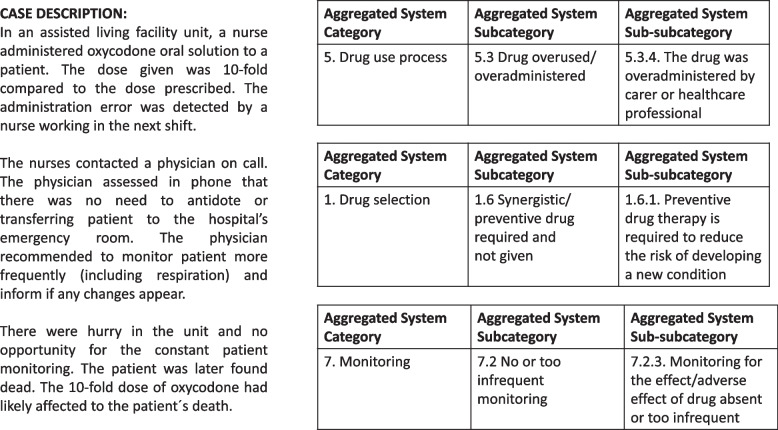
An example of how MEs were identified from the case descriptions (*n* = 58) and categorized with Basger et al.’s aggregated DRP classification system [21]

It was possible to classify all MEs according to Basger et al. [21]. In total, 100 MEs were identified from the cases (*n* = 58) (Table 2). In 53% (*n* = 31) of the cases, more than one ME was identified (mean number of MEs, 1.7 per case). Figure 3 presents an example of how MEs were identified and categorized from the case descriptions. A small proportion (8%, *n* = 8) of the MEs identified from the reports were classified in the “Other” category (a cause that cannot be classified into one of Basger’s et al.’s eight categories, Category 9, Table 2). The”Other” category included the following MEs (*n* = 8):

1. dispensing errors in a community pharmacy (*n* = 1)
2. documenting errors related to medication administration (e.g., no information available if the medication had been administered or in which dose, *n* = 5)
3. prescribing errors in which cessation of the medication was carried out inappropriately (*n* = 1)
4. dispensing error in the care unit detected before reaching the patient (near miss, *n* = 1).

The identified MEs (*n* = 100) fell into all nine main categories of Basger et al.'s classification, 21/33 (64%) of the subcategories and 21/58 (36%) of the sub-subcategories (Table 2). Most of the MEs (*n* = 89, 89%) were categorized into the following six main categories: “Drug selection” (*n* = 22, 22%), “Drug use process” (*n* = 18, 18%), “Monitoring” (*n* = 14, 14%), “Dose selection” (*n* = 13, 13%), “Treatment duration” (*n* = 11, 11%) and “Logistics” (*n* = 11, 11%). Subcategory and sub-subcategories were used for all MEs if there were such defined in Basger et al.´s classification system.

## Discussion

The present study suggests that the aggregated DRP classification system by Basger et al. [21] can be applied to categorizing severe MEs. To our knowledge, this was the first international study to pilot test the applicability of a DRP classification system for this purpose. Further studies are needed to confirm our findings. Because of its authoritative nature, Valvira’s data had comprehensive qualitative and narrative documentation on the ME cases that enabled us to identify reliably multiple MEs and their causes in each case.

As Valvira’s data included both severe and non-severe MEs in different social and healthcare settings, our study indicated that the DRP classification might be applied to MEs with different levels of harm occurring in different care settings. Still, there were some challenges in using aggregated DRP classification system for all MEs included in our data. This concerns especially the potential limitation of not being able to categorize all MEs prevented before they reached the patient (i.e., near misses). With severe MEs, this situation is not usually an issue as they typically reach the patient and cause some problems or harm to the patient. However, this limitation of DRP classification can become a major challenge when classifying MEs that are potential (Fig. 1.). It would be problematic if the same classification system would not apply to near miss events as usually the ME data consists of all kinds of ME, ranging from near misses to most fatal ones. As there was only one near miss case in our study material, there is a need for further research with more extensive data acquired from other ME sources, such as ME reporting systems commonly used in healthcare organizations.

With Basger et al.’s aggregated DRP classification system, we were able to categorize both the problem (ME) and its cause. Subgroups and sub-subgroups of causes were seen to be useful in the categorization of the causes in a way that provides detailed information about the incident for ME prevention and risk management purposes from the systems approach, although further research is needed on ME categorization. Already existing ME classification systems could be further developed to include similar subgroups of error causes as the Basger et al.’s aggregated DRP classification system has. This kind of more detailed error cause categorization has been already experimented within e.g., a medical error study that used the WHO International Classification for Patient Safety [34]. Still, the strength of Basger et al.’s aggregated DRP classification system is that it is readily available and specifically designed for risk management in the medication use process. There is a need to further optimize ME classification so that it will provide enough information about error causes and contributing factors.

Having as much information as possible is a benefit when developing medication safety based on reported errors, especially severe ones [17, 18]. For example, prescribing error (as an outcome) can mean anything starting from the wrong drug or dose selection to duration of the medication treatment [35]. In our previous study with the same Valvira data as used in the present study, we were able to identify the most common ME types but were using an outcome-based ME taxonomy [17]. By this present analysis of the same data using Basger et al.’s aggregated DRP classification system, we were able to describe and understand the MEs and causes contributing to the incidents more in detail. Another strength of using Basger et al.’s aggregated DRP classification system is that it indicates whether the MEs were caused by healthcare professionals or by the patient.

Even though we found Basger et al.’s aggregated DRP classification system to be potential for categorizing severe and non-severe MEs, it could be further optimized for classifying MEs. Basger et al.’s aggregated DRP classification system had many subcategories that did not have any sub-subcategories to describe the cause for the problem (16/58). As we had rich qualitive data and were able to identify the cause(s) for the ME in most cases, we could have been able to add some sub-subcategories to the classification system (e.g., why too low medication dose was prescribed). Additional sub-subcategories could produce more information about the causes and contributing factors. On the other hand, they may increase the complexity of the classification system [21].

It became evident that we were not able to describe all contributing factors identified that caused the error by using the aggregated DRP classification system. For example, in a ME case presented in Fig. 3, we identified many more contributing factors than we were able to classify with the classification system (e.g., two different oxycodone concentrations in use, no double-checking procedures, an inexperienced physician who did not know the possibilities and resources for patient monitoring in assisted living facility unit). This result indicates that Basger et al.’s aggregated DRP classification system in its current form does not alone meet the need for comprehensively categorizing a wide range of factors contributing to severe MEs. Therefore, it is important to recognize the limitations of the DRP classification system and use other methods to supplement the understanding of the multiple causes and factors contributing to MEs.

Even though the aggregated DRP classification system helped to describe MEs and their causes in more detail, we are still lacking tools to describe complex medication errors. Severe errors often consist of multiple errors that are chained (e.g., Fig. 3) [16, 17, 31, 32, 36]. It is challenging to make these error chains visible using only structured classification, although there are some successful examples [16]. But in terms of learning in-depth from severe MEs and complex error cases, qualitative analysis (e.g., root cause analysis or causal tree analysis) can still be seen as more informative, because these tools enable the description of multiple error chains and the contributing factors in the medication use process [37, 38].

All kinds of classification systems need to have an “other” category for miscellaneous issues because classification systems can rarely be completely comprehensive. The prevalence of using this category has been even higher in the studies using different DRP classification systems for DRPs than in our study with MEs [19]. Our data contained documenting errors at the administration phase that we were not able to categorize using Basger et al.’s aggregated DRP classification system. Documenting errors have potential to cause DRPs and thus harm the patient; therefore, they should be added to the classification system [39, 40]. They could be categorized under the main category “Logistics.” At all, the main category “Logistics” could be renamed because some of its sub- and sub-subcategories relate to other than purely logistic issues, such transcribing errors (Category 6.2) and errors in drug selection (Category 6.3) (Table 2). We also found that the classification system was missing an explicit DRP category for problems in medication cessation and implementing phased dose changes. Both these subcategories are important from the DRP and ME perspective.

Furthermore, we noticed that the classification system should be developed from the pharmacists’ perspective to better consider risks related to the preparation and dispensing phases of the medication use process, as well as counseling and educating medicine users. All these situations are potential causes for errors and causes for DRPs [41, 42]. The classification system should distinguish between transcribing errors that occur in medication reconciliation and the transcribing phase. Basger et al. [21] themselves noticed that DRPs associated with care transfers may require a taxonomy of its own, because these problems may be challenging to describe using only the DRP classification system. Indeed, the medication discrepancy taxonomy (MedTax) has been developed few years after Basger et al. published their aggregated DRP classification system [43].

### Limitations

Basger et al.’s aggregated DRP classification system was seen potential for classifying the severe MEs in our study, but more studies are needed using other ME incident data from other reporting systems to confirm our findings. Although our data included all ME cases investigated by Valvira within a five-year period, the data had quite limited number of cases. This problem concerns particularly the number of severe MEs which were the special focus of this study. A larger number of severe ME cases in our data may have allowed us to generate more specific information about the applicability of the DRP classification system. The available data set was large enough to conclude whether DRP classification is in principle suitable for classifying MEs. Our data was rich and extensive, qualitative and narrative in nature. Thus, it was suitable for the qualitative analysis, even though primarily collected for authoritative purposes. The data enabled us to identify many MEs and their causes. Our rich data may also be considered as a limitation when comparing our results with the results of other studies that may have used data with less information about the ME incidents.

In our study, one researcher made the classification and only in cases where difficulties were encountered in the categorization, another researcher was consulted, and final classification decided as consensus. Although the aim of this study was not to validate the DRP classification system, the lack of more thorough validation of the classification process by two independent researchers can be seen as a limitation. Still, our study provides promising preliminary results for suitability of using DRP classification system for classifying and analyzing especially severe MEs. More research is needed to evaluate the applicability and utilization of DRP classification systems for classifying and analyzing ME data of different types and levels of harm, particularly data for understanding severe MEs and their contributing factors.

## Conclusions

Our study suggests that the aggregated DRP classification system with some modifications could be used for analyzing and describing MEs and their causes, especially severe MEs. This finding aligns with the current definition framework in the study which defines that DRPs can be caused by MEs. Using a cause-based DRP classification system produces additional information which is essential for understanding why the MEs happened and how to prevent such MEs in future. This information is particularly valuable with severe MEs that are a priority to prevent. Because severe MEs are often complex processes including several errors and contributing factors, additional means to analyze the incidents are also needed. Further studies using different ME data are needed to validate our preliminary findings and learn more how DRP classification systems could be utilized in learning from errors and their contributing factors.

## Data Availability

The data that support the findings of this study are available from Valvira but restrictions apply to the availability of these data, and are not publicly available.

## Abbreviations

ME: Medication error
DRP: Drug-related problem
ADE: Adverse drug event
ADR: Adverse drug reaction
